# Preoperative anthropomorphic and nutritious status and fistula risk score for predicting clinically relevant postoperative pancreatic fistula after pancreaticoduodenectomy

**DOI:** 10.1186/s12876-020-01397-7

**Published:** 2020-08-08

**Authors:** Tomoyuki Abe, Hironobu Amano, Tsuyoshi Kobayashi, Keiji Hanada, Minoru Hattori, Masahiro Nakahara, Hideki Ohdan, Toshio Noriyuki

**Affiliations:** 1 Department of Surgery, Onomichi General Hospital, 1-10-23, Onomichi, Hiroshima, 722-8508 Japan; 2 Department of Gastroenterological and Transplant Surgery, Graduate School of Biomedical and Health Sciences, Hiroshima University, Hiroshima, Japan; 3 Department of Gastroenterology, Onomichi General Hospital, Hiroshima, Japan; 4 Advanced Medical Skills Training Center, Institute of Biomedical and Health Sciences, Hiroshima University, Hiroshima, Japan

**Keywords:** Fistula risk score, Pancreaticoduodenectomy, Postoperative pancreatic fistula, Skeletal muscle index, Visceral adipose tissue area

## Abstract

**Background:**

Postoperative pancreatic fistula (POPF) is a life-threatening postoperative complication. The aim of this study was to evaluate the efficacy of the fistula risk score (FRS) and preoperative body composition factors for predicting the occurrence of clinically relevant POPF (CR-POPF) after pancreaticoduodenectomy (PD).

**Methods:**

In this study, 136 consecutive patients who underwent PD between 2006 and 2018 were enrolled. The risk factors of CR-POPF (grades B and C) were analyzed. Preoperative visceral adipose tissue area (VATA), skeletal mass index (SMI), and subcutaneous adipose tissue area (SATA) were calculated from computed tomography data.

**Results:**

The overall 30-day mortality and morbidity rates were 0.7 and 38%, respectively. The incidence rates of grade B and C CR-POPF were 27 and 4%, respectively. A univariate analysis revealed that male sex, habitual smoking, prognostic nutritional index (PNI) < 45, VATA ≥90, VATA/SATA ≥0.9, VATA/SMI ≥ 1.4, and FRS > 4 were significantly associated with the incidence of CR-POPF. A multivariate analysis revealed that PNI < 45, VATA/SMI ≥ 1.4 and FRS > 4 were the independent risk factors of CR-POPF.

**Conclusions:**

Preoperative anthropomorphic imbalance, PNI, and FRS were independent risk factors for CR-POPF. Patients with high-risk factors should be closely monitored during the postoperative period.

## Background

Regardless of surgical technique and perioperative management after patients undergo pancreatic resection, the postoperative complication rate remains high at 20 to 50% [1–3]. Among the postoperative complications, severe POPF remains of utmost concern as it can result in massive bleeding. Various risk factors of postoperative pancreatic fistula (POPF) have been identified, including a narrow main pancreatic duct, soft pancreatic texture, perioperative blood transfusion, high body mass index (BMI), and sarcopenia [4–8]. Callery et al. reported that a simple 10-point Fistula Risk Score (FRS) evaluated during pancreaticoduodenectomy (PD) and calculated on the basis of gland texture, pathology, pancreatic duct diameter, and intraoperative blood loss, accurately predicts POPF [9].

In 2016, the International Study Group on Pancreatic Fistula (ISGPF) redefined POPF [10] and recognized its prevalence at 13–30% [1, 4, 5]. Sarcopenia is defined as having a skeletal mass 2 standard deviations lower than the mean for healthy young adults [11, 12]. Skeletal mass is calculated using computed tomography (CT) data. Sarcopenia affects muscle function and is strongly associated with both short-term outcomes and long-term prognosis [7, 11]. Recent studies reported that an abundant visceral adipose tissue area (VATA) and a high ratio of VATA to skeletal muscle was associated with postoperative complications [13, 14]. Increased adipose tissue and sarcopenia deteriorate the host’s immune system, increasing their susceptibility to postoperative complications such as POPF [7, 8]. The aim of this study was to determine the efficacy of using the approach to predicting potential risk factors of CR-POPF from preoperative CT using PNI and FRS scores.

## Methods

### Patients

A total of 136 patients who underwent PD between 2006 and 2018 at the Department of Surgery, Onomichi General Hospital, were enrolled in this study. Patients who received palliative surgery were excluded from the study. Clinical and pathological data and preoperative CT findings were collected. Patients with pancreatic ductal adenocarcinoma, adenocarcinoma of the papilla, extrahepatic cholangiocarcinoma, intraductal papillary-mucinous carcinoma, and chronic pancreatitis were considered eligible. The study was approved by the local institutional review board (OJH-201642), and written informed consent was obtained from all the patients.

### Preoperative CT analysis of body composition

**Fig. 1:**
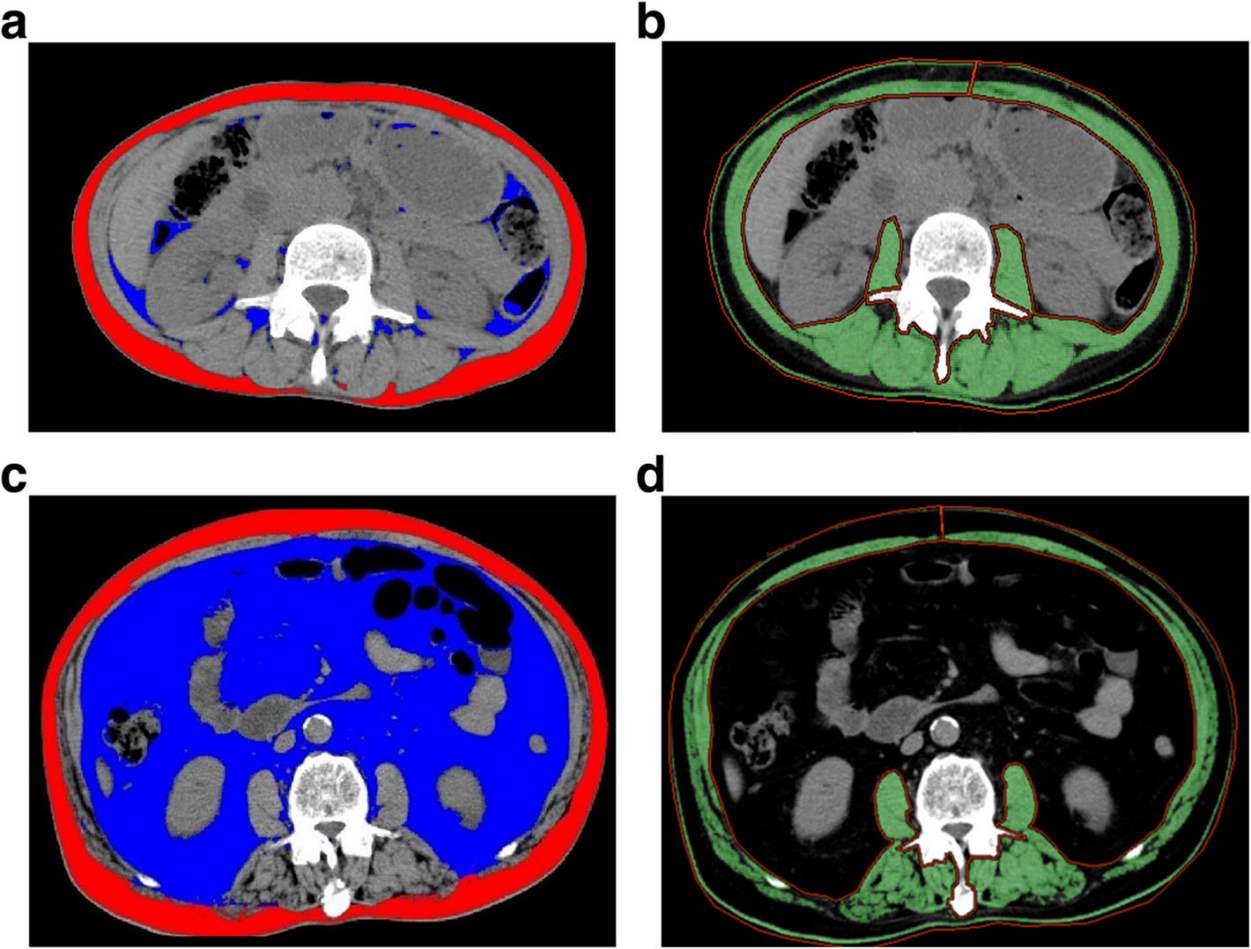
Computed tomography (CT) images at the third lumbar vertebra level. The visceral adipose tissue area (VATA) is highlighted in blue, while the subcutaneous adipose tissue area (SATA) is highlighted in red. Areas of total skeletal muscle is highlighted in green. **a**, **b** CT image of a patient with low VATA/skeletal muscle index (SMI; VATA, 8.0 cm^2^/m^2^; SATA, 43.8 cm^2^/m^2^; SMI, 40.5 cm^2^/m^2^; VATA/SMI, 0.20). **c**, **d** CT image of a patient with high VATA/SMI (VATA, 359.0 cm^2^/m^2^; SATA, 125.2 cm^2^/m^2^; SMI, 42.5 cm^2^/m^2^; VATA/SMI, 8.45)

Eight weeks prior to surgery, all the patients underwent a preoperative evaluation using non-enhanced and enhanced multi-detector CT (Discovery CT 750 HD, GE Healthcare, Milwaukee, WI, and Sensation 16, Siemens, Forchheim, Germany, respectively), with a section thickness of 2.5–5 mm. The CT slices at the third lumbar vertebra (L3) level were analyzed using Advantage Workshop 4.5 (GE Healthcare) and Virtual Place Fujin (AZE Ltd., Tokyo, Japan). The body composition assessment included calculations of the visceral adipose tissue area (VATA), subcutaneous adipose tissue area (SATA), and skeletal muscle area. CT analysis was performed by trained investigators who were blinded to the patients’ characteristics and clinical outcomes (HY, MY). The tissue Hounsfield unit (HU) thresholds were as follows: − 29 to 150 HU for the skeletal muscle area, − 190 to − 30 for the SATA, and − 150 to − 50 for the VATA. Skeletal muscle area was defined at the L3 level and included the psoas major and minor, paraspinal muscles (i.e., erector spinae and quadratus lumborum), and abdominal wall muscles (i.e., transversus abdominus, external and internal obliques, and rectus abdominis). The skeletal muscle area was normalized for height in meters squared (m^2^) to obtain the skeletal muscle index (SMI). The VATA/SMI ratio was defined as high if ≥1.4 and low if < 1.4 (Fig. 1). The cutoff value was estimated using a receiver-operating characteristic (ROC) curve analysis.

### Definition of sarcopenia and pancreatic fistula

Sarcopenia was defined as follows: in men, a SMI of < 43 cm^2^/m^2^ with a BMI of < 25 kg/m^2^ or a SMI of < 53 cm^2^/m^2^ with a BMI of 25 kg/m^2^; and in women, a SMI of < 41 cm^2^/m^2^ [13]. Pancreatic fistula was defined in accordance with the current ISGPF criteria [10], and only fistulas of grades B and C were considered.

### Surgical procedure and postoperative care

Surgery type was selected on the basis of tumor location. Subtotal stomach-preserving PD was routinely performed in the patients. Lymph nodes near the anterior and posterior surfaces of the pancreatic head were resected en bloc, and nodes of the superior mesenteric artery were resected en bloc when technically possible. The pancreatic stump was sutured using two-layer sutures end to end with the invagination of the jejunum. End-to-side hepaticojejunostomy was performed 5 cm distal to the pancreaticojejunostomy followed by side-to-side gastrojejunostomy. A round drain was positioned near the pancreatic anastomosis; and a flat drain, near the biliary anastomosis. Epidurals and non-steroid anti-inflammatory agents were routinely used during the procedure.

The patients with CR-POPF received total parenteral nutrition and octreotide (Sandostatin by Novartis, Rueil Malmaison, France), and a percutaneous drain was inserted under radiological guidance when the collection of infected fluid was suspected on CT.

### Statistical analysis

Continuous variables were reported as medians and analyzed using the nonparametric Mann-Whitney *U* test. Categorical variables were compared using the Fisher exact test. Logistic regression was used to model categorical outcomes. Variables that showed statistically significant associations in the univariate analysis were entered into a multivariate logistic regression model. Differences between the results of the comparative tests were considered significant if a two-sided *p* value of < 0.05 was obtained. An FRS of > 4 was defined as high on the basis of the ROC curve analysis (Table 1). ROC curve analysis was applied to determine the cutoff values for the variables including PNI, VATA, SATA, SMI VATA/SMI, and VATA/SATA, which were determined to be independent risk factors. All the statistical analyses were performed using SPSS version 22 (IBM Corp., Armonk, NY).

**Table 1:** Fistula risk score for the prediction of clinically relevant pancreatic fistula after pancreatoduodenectomy

Risk factor	Parameter	Points^a^
Gland texture	Firm	0
	Soft	2
Pathology	Pancreatic adenocarcinoma of pancreatitis	0
	Ampullary, duodenal, cystic, and islet cell	1
Pancreatic duct diameter, mm	≥5	0
	4	1
	3	2
	2	3
	≤1	4
Intraoperative blood loss, mL	≤400	0
	401–700	1
	701–1000	2
	> 1000	3

^a^Total 0 to 10 points

## Results

### Patient characteristics

The median age of all the enrolled patients was 71 years (range, 35–86 years). Of the 136 patients enrolled, 94 were male and 42 were female. The overall 30-day mortality and morbidity rates were 0.7% (1/136 patients) and 38% (51/136 patients), respectively. The incidence rates of grade B and C POPF were 27.2% (37/136 patients) and 3.7% (5/136 patients), respectively. The most common pathological diagnosis was pancreatic tumor, followed by tumors in the papilla of Vater and extrahepatic bile duct. Preoperative sarcopenia was diagnosed in 53 patients (39%). The patients’ characteristics are shown in supplemental Table 1. The median VATA and SATA were 103.7 and 100.7 cm^2^/m^2^, respectively. The median score for FRS calculation was 4 (range, 0–9).

### Risk factors of POPF

In the univariate analysis of the risk factors of POPF after pancreatectomy, male sex, habitual smoking, PNI < 45, VATA ≥90, VATA/SATA ≥0.9, VATA/SMI ≥ 1.4, and FRS > 4 were identified to be significantly associated with POPF (Table 2). The multivariate analysis results indicated that PNI < 45, VATA/SMI ≥ 1.4 and FRS > 4 score were independent risk factors of POPF.

**Table 2:** Univariate and multivariate analyses of risk factors of postoperative pancreatic fistula after pancreatectomy

Variable	Univariate analysis		Multivariate analysis			
	Non-POPF (*n* = 94)	POPF (*n* = 42)	*p* Value	Hazard ratio	95% CI	*p* Value
Age ≥ 75 years	35 (37%)	12 (29%)	0.339			
**Male**	**60 (64%)**	**34 (81%)**	**0.048**	1.195	0.286–4.990	0.807
BMI ≥ 25 kg/m^2^	14 (15%)	11 (26%)	0.150			
Diabetes mellitus	22 (23%)	5 (12%)	0.163			
**Habitual smoking**	**43 (45%)**	**28 (67%)**	**0.027**	2.571	0.938–7.051	0.067
Habitual alcohol consumption	37 (39%)	22 (52%)	0.193			
Preoperative biliary drainage	34 (36%)	12 (29%)	0.437			
T-Bil ≥ 1 mg/dL	67 (71%)	28 (67%)	0.686			
Alb ≥3.5 mg/dL	66 (70%)	34 (81%)	0.290			
CRP ≥ 1 mg/dL	19 (20%)	9 (21%)	1.000			
**PNI < 45**	**51 (54%)**	**31 (74%)**	**0.037**	**2.659**	**1.045–6.767**	**0.040**
Sarcopenia	40 (43%)	13 (31%)	0.254			
**VATA ≥ 90 cm2/m2**	**39 (41%)**	**32 (76%)**	**0.0002**			
SATA ≥88 cm2/m2	55 (59%)	28 (67%)	0.448			
SMI < 42 cm2/m2	36 (38%)	10 (24%)	0.099			
**VATA/SATA ≥ 0.9**	**45 (48%)**	**32 (76%)**	**0.003**	0.712	0.180–2.809	0.627
**VATA/SMI ≥ 1.4**	**52 (55%)**	**39 (93%)**	**0.0004**	**14.712**	**3.064**–**70.650**	**0.001**
**FRS high score (> 4 score)**	**36 (38%)**	**30 (71%)**	**0.006**	**4.820**	**1.960**–**11.855**	**0.001**
Pancreatic adenocarcinoma or pancreatitis	45 (48%)	24 (57%)	0.357			
**Soft pancreatic texture**	**42 (45%)**	**30 (71%)**	**0.005**			
**Pancreatic duct size < 3 mm**	**33 (35%)**	**28 (67%)**	**0.0011**			
Estimated blood loss > 701 mL	47 (50%)	22 (52%)	0.849			

Abbreviations: *Alb* albumin; *Amy* amylase; *BMI* body mass index; *CI* confidence interval; *Cr* creatinine; *CRP* C-reactive protein; *POD* postoperative day; *POPF* postoperative pancreatic fistula; *PNI* prognostic nutritional index; *SATA* subcutaneous adipose tissue area; *SMI* skeletal muscle index; *T-Bil* total bilirubin; *VATA* visceral adipose tissue area; *WBC* white blood cells

Variables in bold show statistically significant association (*p* < 0.05). All the variables are expressed as number (percentage)

## Discussion

The results of our study demonstrate that high VATA/SMI, PNI < 45 and high FRS score were independent risk factors of POPF after PD. Patients with three risk factors were significantly more likely to have CR-POPF after PD. Previous reports that examined the relationship between sarcopenia and POPF focused only on skeletal muscle mass and visceral adipose tissue [8, 13, 15]. Sandini et al. reported that a high visceral adipose tissue-to-skeletal muscle ratio was a determinant of major postoperative complications after PD for malignancies [14]. Sui et al. reported that sarcopenia was not related to POPF, and patients with non-sarcopenia were strongly associated with POPF [16]. Another report showed that sarcopenia was an independent risk factor of POPF [7]. BMI, which is mainly a reflection of obesity, has been consistently reported as a risk factor of POPF [4]. Considering these reports, a close evaluation of body composition along with evaluations of BMI and sarcopenia could be effective for predicting POPF. Therefore, our VATA/SMI metric may be useful for a more precise evaluation of visceral fatty tissue and skeletal muscle imbalance. Some observations in this present study require clarification with respect to our higher CR-POPF rate than previously reported. First, there was a relatively high rate of soft pancreas and narrow main pancreatic size < 3 mm. Twenty-seven patients (60%) among those with PDAC were diagnosed with the disease at less than the pTNM stage I. The early detection of pancreatic cancer is correlated with long-term survival; however, obstructive pancreatitis caused by tumor progression did not occur in these patients. This could have increased the CR-POPF, even in patients with PDAC. Second, in the reconstruction, the pancreatic stump was sutured using two-layer sutures end to end with the invagination. Due to the relatively high rate of POPF, our reconstruction approach was recently changed to modified Blumgart anastomosis.

The underlying mechanism that links body composition and POPF still needs to be elucidated. Lutz et al. showed that the development of visceral adipose tissue is associated with an elevated production of proinflammatory molecules such as leptin, chemerin, resistin, tumor necrosis factor-α, interleukin (IL) 1, and IL-6 [17]. These proinflammatory cytokines suppress the host immune system and impede wound healing, thereby increasing susceptibility to infectious complications. Another line of thought suggests that in cancer patients, visceral adipose tissue may further disrupt certain catabolic pathways that promote the multimodal development of sarcopenia [18]. Sarcopenia itself may develop as a result of aging, catabolic disorders, cancer, and deficient nutrition, as well as impeded host immune activity through suppressed production of IL-15 [19]. Skeletal muscle tissue produces high levels of IL-15, which normally prevents muscle protein degradation and contributes to the regulation of adipose tissue formation and differentiation. The negative feedback induced by pro-cytokines likely plays a key role in the development of POPF after pancreatectomy.

From a surgical point of view, the presence of abundant visceral adipose tissue may interfere with anastomosis creation, which could result in increased intraoperative bleeding and operation time. Perioperative blood loss should be minimized to protect the patient’s immune system. In addition to high VATA, we determined other risk factors of POPF from pathological findings and postoperative clinical data.

The present multivariate analysis identified that the most effective predictor of CR-POPF was VATA/SMI. High BMI has a well-known association with obesity and surgical complications [20, 21]. Patients with a high proportion of body fat frequently have comorbidities such as diabetes mellitus, hypertension, and chronic heart failure. Obesity rates vary worldwide with low rates in Asia and high rates in America and Europe. Obesity status and BMI are both calculated using a weight-to-height ratio without a detailed consideration of body composition. In fact, the same BMI can be obtained with a large proportion of either total body adipose tissue or total muscle mass. Therefore, preoperative VATA and SMI assessments can provide reliable parameters for the prediction of POPF development and are advantageous because such evaluations could be applied worldwide.

Preoperative nutritional support and rehabilitation programs to decrease the risk of POPF are often implemented for patients with sarcopenia and poor nutritional status. Kaido et al. reported a positive impact of nutritional therapy on prognosis after living-donor liver transplantation especially in patients with sarcopenia [22]. However, multidisciplinary approaches will be required to prevent POPF in patients at high risk.

Several limitations of our study should be mentioned. First, this was a single-institution study, and the data were analyzed retrospectively. In addition, only Asian patients were included. Further studies are required to assess the feasibility of VATA/SMI, PNI, and FRS in predicting CR-POPF development.

## Conclusions

In conclusion, our study demonstrates that preoperative body composition, defined by both visceral adipose tissue and skeletal muscle, are strong predictors of POPF incidence after pancreatectomy. Assessment of preoperative body composition is easy to perform, and reliable measurements can be obtained. Clinicians should closely monitor patients with a PNI < 45 and VATA/SMI ≥ 1.4.

## Supplementary information

**Additional file 1: Table 1.** Clinicopathological data.

## Abbreviations

Alb: Albumin
Amy: Amylase
BMI: Body mass index
CI: Confidence interval
CR-POPF: Clinically relevant postoperative pancreatic fistula
Cr: Creatinine
CRP: C-reactive protein
FRS: Fistula risk score
HU: Hounsfield unit
ISGPF: the International Study Group on Pancreatic Fistula
PD: Pancreaticoduodenectomy
POD: Postoperative day
POPF: Postoperative pancreatic fistula
PNI: Prognostic nutritional index
ROC: Receiver-operating characteristic
SATA: Subcutaneous adipose tissue area
SMI: Skeletal muscle index
T-Bil: Total bilirubin
VATA: Visceral adipose tissue area
WBC: White blood cells
