# Supplementary material for: Preoperative anthropomorphic and nutritious status and fistula risk score for predicting clinically relevant postoperative pancreatic fistula after pancreaticoduodenectomy
Source: BMC Gastroenterol. 2020 Aug 8;20:264. doi: 10.1186/s12876-020-01397-7 (PMC7414683; doi:10.1186/s12876-020-01397-7)
Supplement: Supplementary file 1 — Additional file 1: Table 1. Clinicopathological data. [file 12876_2020_1397_MOESM1_ESM.doc]

Supplemental table 1. Clinicopathological data

| Characteristic | Value (n =136) |
| --- | --- |
| Age, years | 71 (35–86) |
| Male sex | 94 (69.1%) |
| BMI, kg/m^2^ | 22.0 (16.2–33.5) |
| Diabetes mellitus | 27 (19.9%) |
| Habitual smoking | 71 (52.2%) |
| Habitual alcohol consumption | 59 (43.4%) |
| Preoperative biliary drainage | 46 (33.8%) |
| Lymphocyte count /µL | 1540 (288–6208) |
| T-Bil, mg/dL | 0.7 (0.2–13.8) |
| Alb, mg/dL | 3.8 (2.5–4.7) |
| CRP, mg/dL | 0.1 (0.0–8.3) |
| Cr, mg/dL | 0.8 (0.4–4.3) |
| PNI | 46.0 (28.9–63.3) |
| Pathological diagnosis |  |
| Pancreas tumor | 86 (63.2%) |
| Vater’s papilla tumor | 22 (16.2%) |
| Extrahepatic bile duct tumor | 17 (12.5%) |
| Others | 11 (8.1%) |
| R0 resection | 119 (87.5%) |
| Sarcopenia | 53 (40%) |
| SMI, cm^2^/m^2^ | 45.6 (29.7–69.8) |
| VATA, cm^2^/m^2^ | 103.7 (3.8–391.9) |
| SATA, cm^2^/m^2^ | 100.7 (1.9–203.3) |
| Operation time, min | 506 (331–767) |
| Bleeding volume, mL | 707 (108–6588) |
| Pancreatic texture soft | 72 (53.0%) |
| CRP on POD1, mg/dL | 8.5 (3.1–17.7) |
| Drain Amy on POD1, IU/U | 1002.5 (6–97530) |
| POPF grade B/C | 42 (30.9%) |
| Delayed gastric empty | 4 (2.9%) |
| Abdominal abscess | 34 (25.0%) |
| Morbidity | 51 (37.5%) |

Abbreviations: Alb, albumin; Amy, amylase; BMI, body mass index; Cr, creatinine; CRP, C-reactive protein; POPF, postoperative pancreatic fistula; POD, postoperative day; PNI, prognostic nutritional index; SATA, subcutaneous adipose tissue area; SMI, skeletal muscle index; T-Bil; total bilirubin; VATA, visceral adipose tissue area

Continuous variables are expressed as a median (range). Qualitative variables are expressed as a number (%).
